# Coinfection in the host can result in functional complementation between live vaccines and virulent virus

**DOI:** 10.1080/21505594.2022.2082645

**Published:** 2022-06-05

**Authors:** Huai Xu, Andrea L. Krieter, Nagendraprabhu Ponnuraj, Yvette (Yung-Tien) Tien, Taejoong Kim, Keith W. Jarosinski

**Affiliations:** a Department of Pathobiology, College of Veterinary Medicine, University of Illinois at Urbana-Champaign, Urbana, IL, USA; b United States Department of Agriculture, Agricultural Research Service, US National Poultry Research Center, Athens, GA, USA

**Keywords:** complementation, vaccines, herpesvirus, Marek’s disease, transmission

## Abstract

One of the greatest achievements of the last century is the development of vaccines against viral diseases. Vaccines are essential for battling infectious diseases and many different formulations are available, including live attenuated vaccines. However, the use of live attenuated vaccines has the potential for adverse effects, including reversion of pathogenicity, recombination, and functional complementation in the host. Marek’s disease is a serious disease in poultry controlled by live attenuated vaccines that has resulted in increased virulence over the decades. Recombination between circulating field viruses or vaccines is a proposed mechanism for the increase in virulence, however, complementation between vaccines and field strains has not been demonstrated in chickens. Here, we describe functional complementation of vaccines with virulent virus to functionally complement transmission and spread in the host. Using the natural virus-host model of Marek’s disease in chickens, our results show dual infection of target cells in chickens with vaccine and virulent virus providing the opportunity for recombination or complementation to transpire. Interestingly, our controlled results showed no evidence of recombination between vaccine and virulent virus, but functional complementation occurred in two independent experiments providing proof for complementation during natural infection in vaccinated individuals. These results suggest complementation as a potential mechanism for vaccine-mediated viral evolution and the potential for complementation should be taken into consideration when developing novel vaccines.

## Introduction

Evolution of viruses is essential for their survival in nature. Apart from mutations incurred during replication, recombination and complementation are two mechanisms in which viruses can evolve to greater virulence, altered host adaptation, or pathogeneses. The risk of vaccines recombining in hosts to create virulent virus has long been raised and shown to occur in nature using poultry vaccines as a model [1,2]. Complementation – the interaction between viral gene products or gene functions during coinfection that results in increased or decreased yield of parental viruses [3] - by mixed or coinfections has been demonstrated in bacteriophage [4] and plant viruses [5] in nature, but thus far not demonstrated for animal viruses outside of cell culture [6] or non-natural experimental model systems [7]. Using our experimental and natural models of infection and transmission, we tested the potential for recombination or complementation using live vaccines and non-transmissible virulent herpesviruses resulting in transmission.

Two herpesvirus genes are essential for horizontal transmission or interindividual spread of *Gallid alphaherpesvirus* 2 (GaHV2), better known as Marek’s disease virus (MDV), in chickens – namely, glycoprotein C (gC) and the conserved herpesvirus protein kinase (CHPK) [8,9]. MDV causes Marek’s disease in chickens that is characterized by severe clinical symptoms including the development of lymphomas in the viscera and other organs; metabolic dysfunction; and neurological signs like paralysis and ataxia [10]. MDV disseminates in the poultry population similar to dissemination of varicella-zoster virus (VZV) that causes chicken pox and shingles in humans, by shedding from the skin and entering new hosts through the respiratory route [11]. For MDV, infection initiates in pulmonary B lymphocytes and macrophages [12] that presumably transport the virus to lymphoid organs and the skin. In the skin, fully productive replication is completed in feather follicle (FF) epithelial (FFE) skin cells and infectious virus is shed into the environment and the cycle recurs in new hosts.

Marek’s disease is a major economic problem in the poultry industry due to its global distribution and transmissibility. It is controlled through mass vaccination of poultry flocks with live attenuated MDV strains and homologous non-oncogenic avian herpesviruses, including *Gallid alphaherpesvirus* 3 (GaHV3) and turkey herpesvirus (HVT: *Meleagrid alphaherpesvirus*; MeHV1) [13,14]. These vaccines are delivered as live viruses and they are efficient at reducing tumor formation and disease, but do not block dissemination or natural infection of virulent MDV. This has resulted in increasing virulence of MDV over the decades [15]. Importantly, MD vaccines are currently being used as vaccine vectors to deliver antigens of other pathogens, such as avian influenza (AIV), infectious laryngotracheitis (ILTV), Newcastle disease (NDV), or infectious bursal disease (IBDV) viruses [16]. We have recently shown that MD vaccine GaHV3 strain 301B/1 has a similar viral life cycle as MDV in which gC [17] and CHPK [18] are required for natural infection.

Previously, it has been demonstrated that two virulent MDVs can infect the same cell [19]. Here, we asked two questions. First, can live MD vaccines and virulent MDV infect the same cells in the chicken, and second, if so, does this result in recombination or complementation? We found that both MD vaccines and MDV can infect the same cells *in vivo* that resulted in functional complementation to facilitate transmission of MDV.

## Materials and Methods

### Ethics statement

All *in vivo* experiments were conducted according to national regulations and ARRIVE guidelines in compliance with approval of the Institutional Animal Care and Use (IACUC) and Biosafety (IBC) Committees at the University of Illinois at Urbana-Champaign (UIUC). UIUC’s animal care facilities and programs are accredited by the Association for Assessment and Accreditation of Laboratory Animal Care (AAALAC). All work meets the requirements of the law (89–544, 91–579, 94–276) and NIH regulations on laboratory animals and are compliant with the Animal Welfare Act, PL 279.

### Animal experiments

Three-day old specific pathogen free (SPF) Pure Columbian (PC) chickens were obtained from the UIUC Poultry Farm (Urbana, IL) and food and water were provided *ad libitum* for all bird experiments. Viruses used in this study have been previously described [17,18,20]. Previously titrated cell-associated MDV and vacMD were diluted and mixed prior to injection into chickens by intra-abdominal inoculation to establish experimental coinfection (Experiment 1, n = 12; Experiment 2, n = 13). Following inoculation, viruses were back tittered and were as follows: For Experiment 1, 2,000 and 7,000 plaque forming units (PFU) of non-transmissible MDV (MDV-NT) and transmissible MD vaccine (vacMD-T), respectively, were inoculated into chicks, while in Experiment 2, 3,000 and 6,000 PFU of MDV-NT and non-transmissible MD vaccine (vacMD-NT), respectively, were used. The higher dose of vacMD used was based on previous studies in which this vaccine requires higher doses that MDV for establishing infection [18,21].

To test natural infection through horizontal transmission, age-matched, naïve contact chickens were housed with experimentally infected chickens for 11 weeks. A total of 8 and 9 contact chickens were used for Experiments 1 and 2, respectively. The number of birds per group is based on a power analysis that relies on a change in transmission from 0% to 25% of contact birds with a standard deviation of 10%. This gives a power score of 1.00 using 8 chickens per group over the course of the experiment.

To test replication and transmission of v9620 and v9628 MDV isolated in Experiments 1 and 2, Experiment 3 was performed. Cell-associated v9620 and v9628 were inoculated into 3-day old PC chickens by intra-abdominal inoculation. Back titers of each virus were 6,500 and 5,750 PFU for v9620 and v9628, respectively. For both viruses, eight chickens were experimentally infected and housed with age-matched sentinel contact chickens (v9620, n = 5; v9628, n = 7) for 9 weeks.

### Immunofluorescence assays and laser scanning confocal microscopy

A mixture of MDV-NT (RB-1B strain) with vacMD-T or vacMD-NT (301B/1 strain) were used to infect chick embryo cell (CEC) cultures prepared with fertilized white leghorn eggs using standard methods [22] during back titration. At 5 days pi, cells were fixed with PFA buffer (2% paraformaldehyde, 0.1% Triton X-100) for 15 min and then washed twice with PBS. Cells were blocked in 10% neonatal calf serum and stained with anti-MDV chicken sera and goat anti-chicken IgY-Alexa Fluor^TM^ 488 secondary antibody (Molecular Probes A11039, Eugene, OR). The virus-induced plaques were observed using an EVOS^TM^ FL Cell Imaging System (Thermo Fisher Scientific) and compiled using Adobe® Photoshop® version 21.0.1.

Coinfected skin/feather tissues were snap-frozen in Tissue Tek®-optimal cutting temperature (OCT) compound (Sankura® Finetek, Torrance, CA) and stored at −80°C. Ten micrometer sections were affixed to Superfrost/Plus slides (Fisher Scientific, Pittsburgh, PA) and counterstained with Hoechst 33,342 (2 µg/ml, Molecular Probes) to visualize nuclei. Images were captured with a Nikon A1 Confocal Laser Microscope with the NIS-Elements C platform and compiled using Adobe® Photoshop® version 21.0.1.

### Monitoring MDV and vacMD in feather follicles (FFs)

To determine when chickens were infected with MDV and vacMD, two flight feathers were plucked from the right and left wings (four total) of experimentally infected chickens weekly beginning at 14 days pi for Experiment 1 and 6 days pi for Experiments 2 and 3. Feathers were monitored in contact chickens starting at 35-, 27-, and 21-days pi for Experiments 1, 2, and 3, respectively. Expression of pUL47eGFP (MDV) and pUL47mRFP (vacMD) was examined using a Leica M205 FCA fluorescent stereomicroscope with a Leica DFC7000T digital color microscope camera (Leica Microsystems, Inc., Buffalo Grove, IL).

### DNA extraction from infected cells and tissues and PCR assays

DNA was extracted from infected FFE cells scraped from FFs or CEC cultures using the QIAamp DNA Mini Kit from Qiagen, LLC (Germantown, MD, USA) according to the manufacturer’s instructions. PCR was performed using DreamTaq Green 2× Master Mix (Thermo Fisher Scientific) and previously described primers to MDV gC and UL13 [23,24]. All PCR reactions were electrophoresed through 0.8% agarose gels and recorded using an Alpha Imager HP (Protein Simple) (Wallingford, CT, USA).

### Virus isolation from chickens

Tumors collected from contact birds #9620 and #9628 positive for MDV in Experiments 1 and 2, respectively, were used for isolation of infecting virus using standard methods [25]. Briefly, spleen tumor tissues were harvested and sieved through a cell strainer and lymphocytes were isolated using Histopaque®-1077 (Sigma-Aldrich) density gradient centrifugation and 2 × 10^4^ cells were seeded onto primary CEC cultures. MDV foci were observed 5 days later and propagated for another two rounds of amplification in CEC cultures before freezing stocks and titrating.

## Results

### Coinfection of non-transmissible MDV (MDV-NT) with MD vaccines (vacMD)

**Figure 1.**
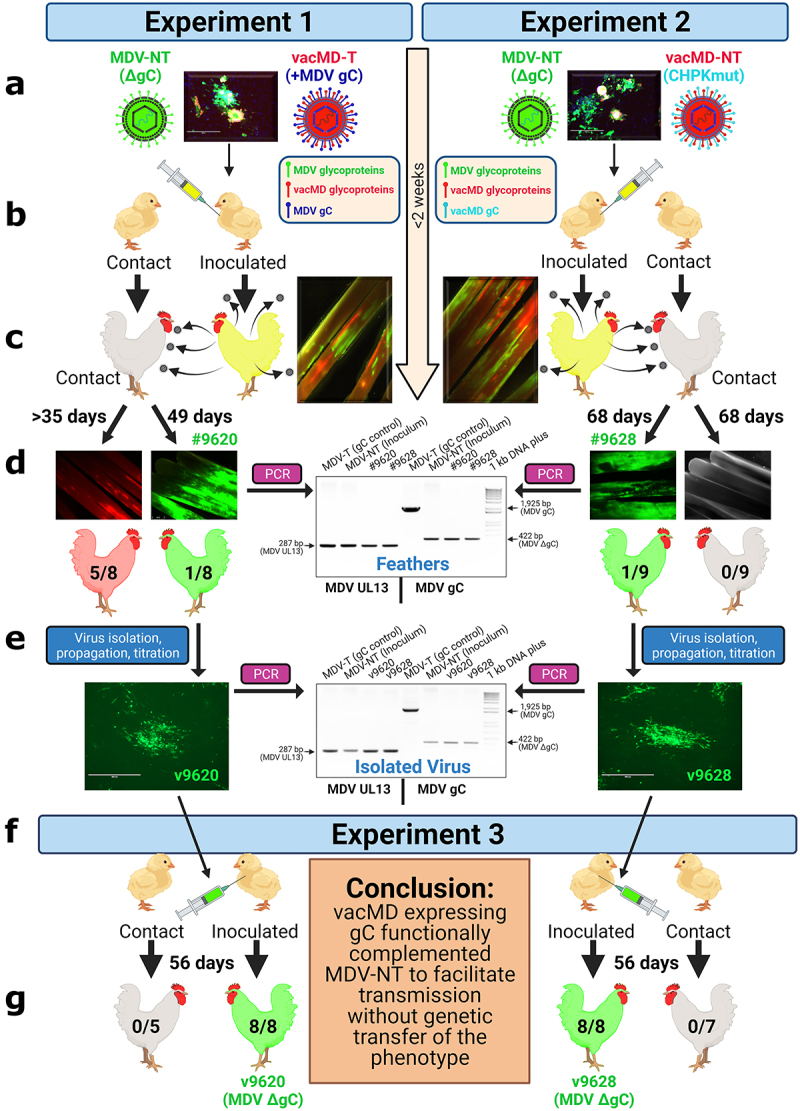
Graphical Abstract: Testing recombination and complementation during coinfection *in vivo*. (a) Chickens were inoculated with a mixture of MDV and vacMD to create a coinfection scenario. (b) Inoculated chickens were housed with uninfected contact chickens. (c) Approximately 2 weeks post-inoculation, chickens infected with viruses are positive in their feathers that was visualized using fluorescent stereomicroscopy. (d) in Experiment 1, five contact chickens were positive for red vacMD, while one chicken (bird #9620) was positive for green MDV based on fluorescence. In Experiment 2, no contact chickens were positive for red vacMD, while one contact chicken (bird #9628) was positive for green MDV. PCR on DNA extracted from infected feathers of birds #9620 and #9628 using MDV specific primers for gC. DNA from the inoculum (MDV-NT) and wild-type MDV (MDV-T) were used as positive controls in which MDV-NT generates a 422 bp product, while MDV-T produces full-length 1,925 bp product encoding MDV gC. PCR for MDV UL13 was used as an internal control for MDV genomes and DNA quality. (e) Viruses isolated from tumors obtained from bird #9620 (v9620) and #9628 (v9628) were propagated for two passages and DNA was used in PCR assays, as was done for DNA from feathers to confirm they remained gC negative. (f) v9620 and v9628 isolated in (E) were inoculated into chickens and chickens were monitored for replication in FFs and MD induction. Contact chickens were housed with v9620- and v9628-infected chickens and remained negative for MDV over 8 weeks confirming both viruses did not gain transmissibility through a genetic component. .

**Figure 2.**
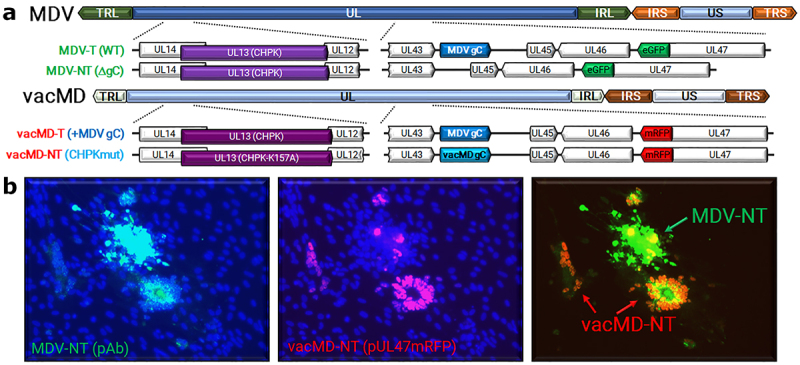
Coinfection of MDV-NT and vacMD in CEC cultures. (a) Schematic representation of transmissible (T) and non-transmissible (NT) MDV and vacMD used in this study. MDV-NT lacks gC (MDV δgc) that is required for transmission [8,9,23]. vacMD was rendered NT through mutation of its CHPK activity by mutation of the invariant lysine (K157A) that is required for transmission [18]. vacMD-T expresses MDV gC in place of its gC [17]. (b) Following inoculation into chickens, the inoculum was back tittered in CEC cultures and shown are representative plaques for MDV-NT and vacMD-NT (vacMD-T not shown). Direct visualization of pUl47mrfp was used to identify vacMD-NT plaques, as indicated by strong nuclear expression as previously shown [17], while expression of MDV pUl47egfp is barely detectable in cell culture [26]; therefore, polyclonal anti-MDV antibody was used to identify MDV-NT plaques with anti-chicken IgY secondary antibody conjugated with AlexaFluor488. Anti-MDV pAb has cross reactivity with homologous vacMD and thus stains both MDV- and vacMD-induced plaques but stains MDV plaques with greater intensity. The intense staining with pAb and negative for pUl47mrfp expression indicates MDV infected plaques, while light staining with pAb and high pUl47mrfp nuclear expression is indicative of vacMD infection.

It has been demonstrated in chickens that two different virulent MDVs can frequently coinfect FFs and dually infect FFE skin cells *in vivo* [19], providing ample opportunity for recombination or complementation events to occur. Here, we sought to address the question of whether MD vaccines can coinfect FFs and dually infect FFE skin cells, and subsequently recombine with, or functionally complement, MDV using transmission as a phenotypic read-out. Using our highly efficient experimental and natural infection model, we tested our hypothesis in two independent coinfection experiments (Figure 1). In Experiment 1, chickens were inoculated with non-transmissible MDV (MDV-NT) and transmissible MD vaccine (vacMD-T), while in Experiment 2, chickens were inoculated with MDV-NT and non-transmissible MD vaccine (vacMD-NT). A summary of viruses used in this study are shown along with plaques formed during coinfection in cell culture showing both viruses present in the mixture (Figure 2). Chickens were inoculated with a mixture of MDV-NT (lacking MDV gC) and vacMD-T (vacMD expressing MDV gC) or vacMD-NT (vacMD that has CHPK mutated) of which all viruses have previously been published for their transmissibility [17,18,20]. MDV-NT lacks gC, required for transmission and expresses enhances green fluorescent protein (eGFP), while both vacMD-T and vacMD-NT express monomeric red fluorescent protein (mRFP) allowing us to differentiate each virus in cell culture and *in vivo*. In order to detect gC, we use vacMD expressing MDV gC that facilitates transmission and enabled us the ability to detect the gC protein using antibodies for downstream detection [17].

After 2 weeks, birds inoculated with MDV and vacMD showed both green and red fluorescence in the feathers indicating both viruses reached the feathers (Figure 1(c)). Over the course of both experiments, most chickens experimentally infected had both viruses present in the feathers and this is summarized in Tables S1 and S2. The relative level of infection representing negative (-), low positive (+), intermediate positive (++), and heavily positive (+++) are shown in Figure S1.

### Superinfection inhibition and coinfection of FFE skin cells in vivo

**Figure 3.**
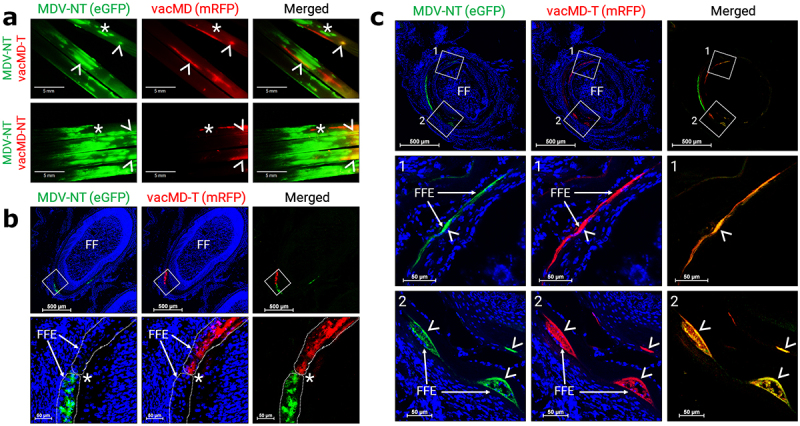
Superinfection inhibition and coinfection of MDV and vacMD in feathers. (a) Coinfected feathers from experimentally infected chickens. Arrowheads (>) indicate coinfected regions where both green MDV and red vacMD are evident in the merged image creating yellow, while asterisks (*) indicate regions where there is a clear delineation of green MDV and red vacMD in proximity suggestive of superinfection inhibition. (b) a representative cryosection of green MDV and red vacMD coinfected FFE skin cells suggestive of superinfection inhibition. The region highlighted with a white square is shown at greater magnification below. Clear delineation of green MDV and red vacMD can be seen and highlighted with dotted line and asterisk (*). (c) a representative cryosection of green MDV and red vacMD coinfected FFE skin cells. Regions 1 and 2 are magnified below and coinfection is clearly observed, indicated by merging of green MDV and red vacMD to generate yellow cells. Nuclei were stained with Hoechst 33342 to better visual cellular structure. Feather follicles (FF) and FF epithelial (FFE) skin cells are labeled in the images with arrows (→).

Superinfection inhibition, where infection of a cell by one virus inhibits infection with a second virus, has been described for many viruses [27–31]. Previously, it has been shown that FFs coinfected with two MDVs exhibit both superinfection inhibition and coinfection of FFE skin cells [19]. To address whether this occurs with MDV and vacMDs, we examined FFs and FFE skin cells for evidence of coinfection using fluorescent microscopy. Like former studies with MDV, evidence of superinfection inhibition could be seen where clearly defined borders between both viruses could be repeatedly seen (Figure 3(a,b)). Dual infection of FFE skin cells was also frequently observed in which both viruses were present within the same cell (Figure 3(a,c)). These results show both superinfection inhibition and coinfection were present in FFE skin cells as was formerly seen for two MDVs [19], the later required for recombination or complementation to materialize.

### Transmission of MDV-NT through complementation by live MD vaccines

Contact sentinel chickens were monitored for natural infection by plucking feathers weekly, as well as evaluated for clinical symptoms of MD. Over the course of Experiment 1, five out of eight contact chickens were naturally infected with vacMD-T (Figure 1(d)), consistent with the ability of vacMD-T to transmit to contact chickens [17]. In Experiment 2, no contact chickens were infected with vacMD-NT, also consistent with the inability of this vaccine to spread in chickens due to the CHPK mutation [18]. Remarkably, in both experiments, one contact chicken was positive for green fluorescence in the feathers suggestive of natural infection with green MDV only (Figure 1(d), Fig. S2). This was confirmed when both birds developed symptoms of MD, including lethargy and visceral tumors following necropsy. All uninfected or vacMD-T infected contact chickens in both experiments remained tumor free.

Since coinfection of the same cell could result in recombination or complementation between MDV and vacMD, we first determined whether MDV-NT incorporated MDV gC (from vacMD-T) or vacMD gC (from vacMD-NT) into its genome through recombination. PCR analysis of DNA collected from FFE skin cells of each contact chicken (#9620 and #9628), as well as virus isolated from these birds confirmed both viruses (v9620 and v9628) lacked the gC gene within the virus genome (Figure 1(e)). Thus, there was no evidence that MDV-NT recombined with vacMD to incorporate gC into its genome. Together, these results show that transmission of MDV-NT in Experiments 1 and 2 was not due to incorporation of MDV or vacMD gC into their genomes.

### Transmission of MDV-NT is not retained in the absence of vacMD

To determine whether v9620 and v9628 MDV gained transmissibility through genetic modifications other than obtaining gC, both isolated viruses were propagated, tittered, and inoculated into chickens and transmission was monitored in Experiment 3 (Figure 1(f)). After two weeks, all inoculated chickens were positive for green fluorescence and ultimately succumbed to MD by 6 weeks post-infection (pi). No contact chickens housed with v9620- or v9628-infected chickens were infected by 8 weeks, confirming both viruses maintained the MDV-NT phenotype (Figure 1(g)). These results confirm v9620 and v9628 isolated in Experiments 1 and 2 did not gain transmissibility through genetic modifications, such as recombination or mutation, that was passed to their progeny.

### Localization of MDV gC with MDV-NT-infected FFE skin cells

**Figure 4.**
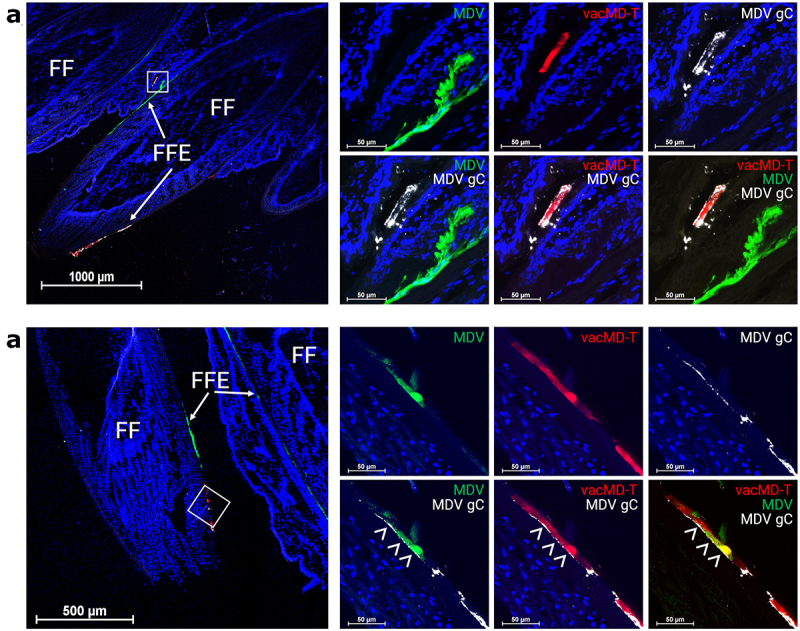
MDV gC expression in coinfected FFs and dual infected FFE skin cells. (a) Cryosection of FFs showing both green MDV and red vacMD individually infecting FFE skin cells. Anti-MDV gC antibody was used to identify MDV gC positive cells. Only vacMD-T showed staining, while green MDV-NT-infected cells were negative. (b) Cryosection of an FF where dual infection with green MDV and vacMD was clear. Anti-MDV antibody stains red vacMD-infected cells, including dual infected “yellow” cells. Feather follicles (FF) and FF epithelial (FFE) skin cells are labeled in the images with arrows (→) and arrowheads (>) indicate MDV gC staining. Nuclei were stained with Hoechst 33342 to better visual cellular structure.

Next, we examined the expression of MDV gC in FFE skin cells from experimentally infected chickens in Experiment 1. Using two representative chickens in Experiment 1, MDV gC was clearly localized to red vacMD-T infected cells using anti-MDV gC antibody in Figure 4(a), while green MDV-infected cells were negative for MDV gC since MDV-NT lacks this gene. Where coinfection is evident in Figure 4(b), MDV gC staining localized with red vacMD-T infected cells and it can be seen (arrowheads) where both green MDV-NT and MDV gC localize to the same cell due to coinfection with vacMD-T. Thus, coinfection with both MDV-NT and vacMD-T provide MDV gC from vacMD-T to functionally complement MDV-NT for transmission in chickens. Most likely, a similar scenario occurred in Experiment 2 in which vacMD gC localized with MDV-NT, however, we lack an antibody to vacMD gC to directly show this scenario. In all, these results show through direct visualization of localization of MDV gC (expressed by vacMD-T) with MDV-NT that could provide functional complementation by coinfection with virulent and vaccine viruses. These results were confirmed in Experiment 3 in which MDV-NT did not transmit in the absence of coinfection with vacMD (Figure 1(g)).

## Discussion

Here, we addressed two overall hypotheses. First, we asked whether live MD vaccines and virulent MDV can infect the same cells *in vivo*. Our results confirmed that coinfection of FFE skin cells with non-transmissible MDV (MDV-NT) and live MD vaccines (vacMD-T or -NT) was evident. Second, we asked whether coinfection of cells could result in recombination or complementation. Using an experimental and natural coinfection model, we showed functional complementation in which non-transmissible MDV (MDV-NT) was able to transmit to chickens in one round of transmission by complementation with vacMD. When both viruses (v9620 and v9628) isolated during Experiments 1 and 2 were introduced into naïve chickens, both viruses failed to transmit in the absence of coinfection with vacMD. The inability to transmit in Experiment 3 showed that recombination or intergenic mutations did not mediate transmission for both v9620 and v9628 in these experiments. Although this is not the first time in which functional complementation has been observed *in vivo*, to our knowledge, this is the first experimental evidence of a live attenuated vaccine coinfecting cells and functionally complementing transmission of a previously non-transmissible virus without genetic transfer during natural infection.

One interesting observation is that in Experiment 1, contact chicken #9620 was only infected with MDV-NT, while vacMD-T was detected in five out of eight contact chickens. It is possible these results were due to viral interference or superinfection exclusion [32]. It is well established that MD vaccines have driven MDV to breakthrough vaccine protection [15] that is linked to increased replication rates [33]. Thus, viral interference or superinfection exclusion could explain why bird #9620 was only infected with one virus if MDV-NT could outcompete, and possibly interfere, with vacMD-T replication during natural infection [32]. In our former studies examining coinfection and superinfection with two MDVs, we found strong superinfection inhibition when one virus was inoculated prior to the second [19]. Thus, viral interference or superinfection exclusion likely played a significant role in our results here and may also help to explain why we only found a single virus in contact chickens infected in Experiment 1 (i.e. 5/8 with vacMD-T and 1/8 with MDV-NT) and none infected with both viruses. Further studies are warranted to address these possibilities.

It is interesting to note that in Experiment 2, where chickens were inoculated with non-transmissible MDV-NT and vacMD-NT, only MDV-NT was able to transmit through complementation of vacMD gC, while we did not observe vacMD-NT transmission through complementation of MDV CHPK. There are few possible reasons for the lack of reciprocal complementation. First, the viral protein required for complementation may not be compatible. That is, we know MDV gC can compensate for vacMD gC using classical gene exchange experiments [17], suggesting both proteins are functionally similar, and the data presented here confirms this. However, it is currently not known whether MDV and vacMD CHPK can compensate for each other during transmission. Thus, the lack of complementation for vacMD CHPKmut by MDV CHPK may be due to incompatible gene functions. Secondly, regardless of whether MDV CHPK can compensate for vacMD CHPKmut, the level of expression of CHPK is considerably lower in cells compared to gC with CHPK being a regulatory protein, while gC a late structural protein [34]. Additionally, since MDV-NT lacks gC, there would be no competition for gC protein expression and incorporation into the viral envelope, whereas both MDV CHPK and vacMD CHPKmut would compete for incorporation into the virion tegument during coinfection. Thus, this would limit the potential for complementation with MDV CHPK into the vacMD virion. Thirdly, the viral components and their interactions required to tegument assembly, including vacMD CHPKmut, most likely have a higher affinity for each other relative to MDV CHPK. In contrast, gC is localized to the viral envelope and incorporated as part of the viral envelope during budding where it would be presumed that less viral specificity is required.

**Figure 5.**
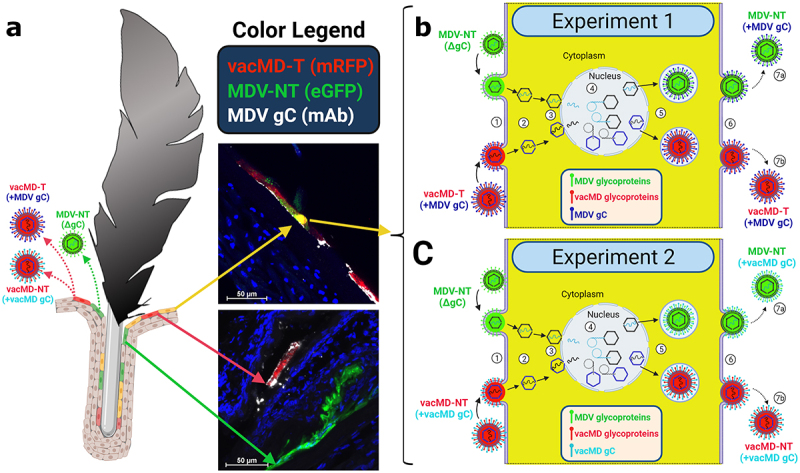
Cartoon depiction of functional complementation *in vivo*. (A) Coinfection of chickens with MDV and vacMD results in FFE skin cells individually infected with each virus (shown as red or green), as well as coinfection in which both viruses are present (shown as yellow). Through normal virus replication in cells, red and green cells would produce progeny of the infecting virus and thus, red cells produce vacMD-T or -NT, while green cells produce MDV-NT. Representative images of red, green, and yellow cells are shown, including staining for MDV gC with mAb (white). (B and C) Basic replication cycle of herpesviruses including (1) entry into cells, (2) uncoating of tegument, (3) docking of nucleocapsid to the nuclear membrane and expulsion of genomic DNA into the nucleus. In the nucleus, (4) viral DNA is duplicated and encapsulated into naked capsids to produce newly generated nucleocapsids that then exit the nucleus (5) where tegumentation and primary envelopment produces virions that egress (6) from the cell as cell-free virus. In dually infected cells, an infinite combination of viral proteins and genomes can be produced if compatible (not shown). Based on the results presented in this report, MDV-NT genomes were packaged into virions in which gC provided from vacMD-T (+MDV gC) or vacMD-NT (+vacMD gC) to generateinfectious MDV able to naturally infect chickens (7a), while the original vacMD was also produced (7b) in each experiment. Thus, transmissible MDV-NT virions would be produced, disseminated into the environment, and naturally infected naïve contact chickens. However, infected contact chickens would be unable to produce transmissible MDV-NT, as shown in Experiment 3, without complementation from coinfection with vacMD.

Figure 5 summarizes the likely scenario that lead to functional complementation in Experiments 1 and 2. Within the FFs, cells infected with only MDV or vacMD, as well as cells dually infected with MDV and vacMD were evident (Figures 3 and 4), consistent with former studies in which superinfection inhibition and coinfection has been observed [19]. It would be predicted that cells infected with only one virus would produce progeny that are phenotypically similar to the infecting virus – that is, MDV-NT, vacMD-NT, and vacMD-T produced MDV-NT, vacMD-NT, and vacMD-T progeny, respectively, as our transmission results show. However, when cells were infected with both green MDV and red vacMD, as evidenced by merging of signals to create yellow cells, viral proteins from both viruses are expressed and can form progeny containing both viruses’ proteins. This is clear examining expression of MDV gC during coinfection with MDV-NT and vacMD-T in Figure 4(b) where FFE skin cells infected with green MDV-NT and red vacMD-T were positive for MDV gC (expressed by vacMD-T) and could be incorporated into MDV particles. The likely outcome of coinfection of a cell by vaccine and virulent virus resulted in MDV particles in which either MDV gC expressed from the vacMD-T genome (Figure 5(b) - Experiment 1) or vacMD gC from vacMD-NT genome (Figure 5(c) - Experiment 2) was provided during virion assembly. The functional requirement of gC for transmission has been demonstrated repeatedly [8,9,17,20,23] and these data amplify the importance of this viral glycoprotein for herpesvirus transmission.

Recombination was not seen in these experiments, but we cannot exclude the potential for recombination in the natural setting. In fact, it was recently shown that recombination occurs in nature between MD vaccines and circulating virulent strains in China [2]. Here, we showed that both MDV and MD vaccines dually infect FFE skin cells during coinfection confirming the dual infection of cells occurs *in vivo* that is prerequisite for recombination or complementation to occur. However, both MDV and MD vaccines establish latency in chickens and can reactivate for the lifetime of the host. For this reason, both complementation and recombination are possible throughout the lifetime of the host potentiating scenarios like what is presented in this report.

The data shown here provide direct evidence that coinfection resulted in complementation between vaccine and virulent viruses during natural infection. This report is not meant to alarm, but these results suggest greater forethought into the design of live vaccines and vaccine vectors in the modern age of a vaccinology. Particularly for the poultry industry in which chickens are vaccinated with mono-, bi- and tri-valent MD vectored vaccines expressing other viral antigens that could potentiate unforeseen consequences if not carefully and thoroughly envisaged. MD vaccines are currently used as multi-valent vaccine vectors to deliver antigens of other pathogens, such as influenza, ILTV, NDV, or IBDV [16]. Usually, the extraneous proteins expressed in these vaccine vectors are envelope and glycoproteins that are highly immunogenic. However, extra precaution and forethought should be employed in the generation of these vaccines with the results presented in this report in which both vaccine and virulent virus dually infected cells, functionally complementing horizontal transmission.

## Supplementary Material

Supplemental Material

## Data Availability

The authors confirm that the data supporting the findings of this study are available with the article and its supplementary materials.
