# Supplementary material for: Coinfection in the host can result in functional complementation between live vaccines and virulent virus
Source: Virulence. 2022 Jun 5;13(1):980–9. doi: 10.1080/21505594.2022.2082645 (PMC9191873; doi:10.1080/21505594.2022.2082645)
Supplement: Supplemental Material [file KVIR_A_2082645_SM1161.zip › supplementary/supp figure.pdf]

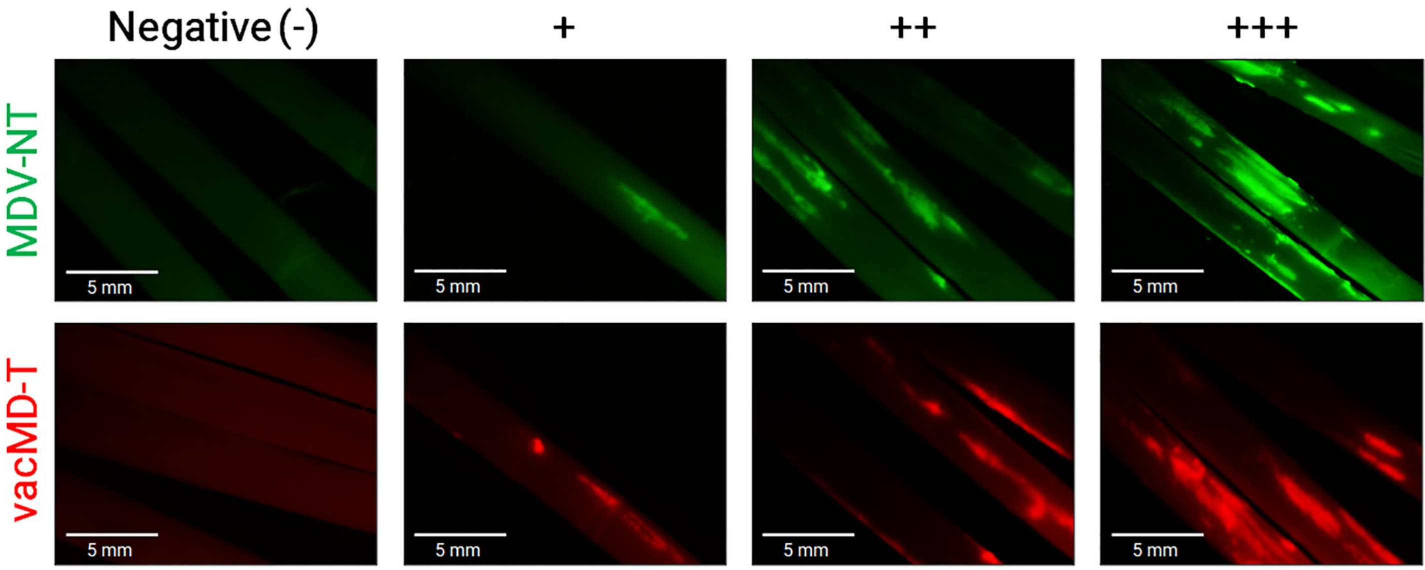

Figure S1. Scoring of infection in feathers. Representative images of scoring for negative and positive feathers infected with MDV-NT and vacMD-T (vacMD-NT not shown).

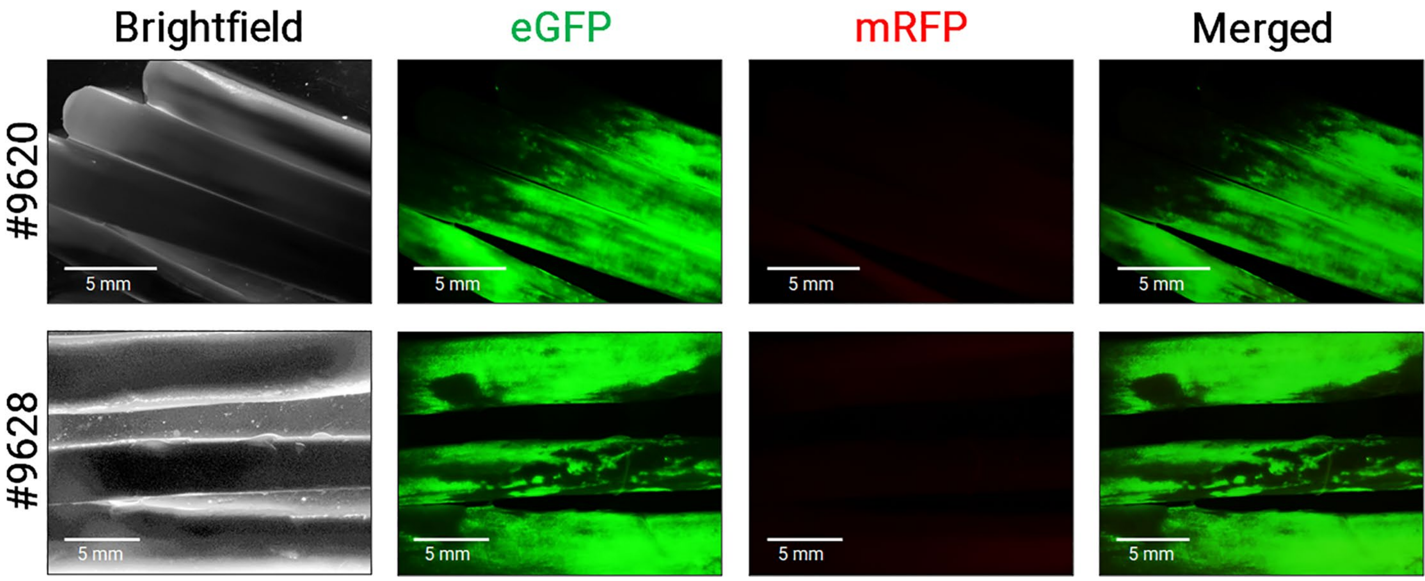

Figure S2. Transmission of MDV-NT. Feathers obtained from birds #9620 (day 49) and #9628 (day 68) with both birds strongly positive for eGFP, presumably green MDV.
