# Supplementary material for: Coinfection in the host can result in functional complementation between live vaccines and virulent virus
Source: Virulence. 2022 Jun 5;13(1):980–9. doi: 10.1080/21505594.2022.2082645 (PMC9191873; doi:10.1080/21505594.2022.2082645)
Supplement: Supplemental Material [file KVIR_A_2082645_SM1161.zip › supplementary/SuppTables.pdf]

Table S1. Coinfection with green MDV-NT + red vacMD-T (Experiment 1).

Infected[A]	Day 14[B]		Day 21		Day 28		Day 35		Day 42		Day 49		Day 56		Day 63		Day 70		Day 77	
Bird #	vacMD	MDV	vacMD	MDV	vacMD	MDV	vacMD	MDV	vacMD	MDV	vacMD	MDV	vacMD	MDV	vacMD	MDV	vacMD	MDV	vacMD	MDV
9528	+[C]	-[D]	++	++	+	+	-	++	+	+++	++	+++	+	+++						
9530	+++	+	++	++	+	+	+	++												
9540	+++	+++	E																	
9541	++	++	++	++	+	+	-	++	+	+++	++	+++	++	++	-	-	-	-	-	++
9594	+++	+++																		
9601	+++	-	++	++	-	+	+	+	+	+										
9605	+++	++	-	++	-	+	+	++	++	++	++	+++	++	++	++	++	++	++	++	++
9606	+++	++	+	++	+	+	++	++												
9608	+++	++	++	++	+	+														
9609	+	-	+	+++	-	+	-	++	-	++										
9610	+++	+	+	+++	+	+	+	+	+	++	+		+							
9615	++	+	-	++	-	-	+	+	+	++	++	+++	++	+++	++	++	+	++	+	+++

Contacts[F]	Day 14		Day 21		Day 28		Day 35		Day 42		Day 49		Day 56		Day 63		Day 70		Day 77	
Bird #	mRFP	eGFP	mRFP	eGFP	mRFP	eGFP	mRFP	eGFP	mRFP	eGFP	mRFP	eGFP	mRFP	eGFP	mRFP	eGFP	mRFP	eGFP	mRFP	eGFP
9620[G]	ND[H]	ND	ND	ND	ND	ND	-	-	-	-	-	+++	-	+++						
9637	ND	ND	ND	ND	ND	ND	+	-	++	-	+	-	+	-	+	-	+	-	+	-
9650	ND	ND	ND	ND	ND	ND	-	-	-	-	-	-	++	-	+	-	++	-	++	-
9639	ND	ND	ND	ND	ND	ND	-	-	-	-	-	-	-	-	+	-	+	-	+	-
9623	ND	ND	ND	ND	ND	ND	-	-	-	-	-	-	-	-	-	-	-	-	+	-
9626	ND	ND	ND	ND	ND	ND	-	-	-	-	-	-	-	-	-	-	-	-	++	-
9636	ND	ND	ND	ND	ND	ND	-	-	-	-	-	-	-	-	-	-	-	-	-	-
9648	ND	ND	ND	ND	ND	ND	-	-	-	-	-	-	-	-	-	-	-	-	-	-

[A]Birds experimentally infected with green MDV-NT and red vacMD-T.
[B]Days after experimental infection.
[C]Relative level of infection of feathers based on fluorescence (see Fig. S1).
[D]Negative for infection based on fluorescence (see Fig. S1).
[E]Blacked out boxes indicate birds were euthanized due to clinical MD symptoms and no longer monitored.
[F]Contact chickens housed with experimentally infected birds to monitor natural infection.
[G]Bird #9620 was positive for eGFP at Day 49 (see Fig. S2).
[H]ND=not done.

Table S2. Coinfection with green MDV-NT + red vacMD-NT (Experiment 2).

Infected[A]	Day 6[B]		Day 13		Day 20		Day 27		Day 34		Day 43		Day 50		Day 56		Day 68	
Bird #	vacMD	MDV	vacMD	MDV	vacMD	MDV	vacMD	MDV	vacMD	MDV	vacMD	MDV	vacMD	MDV	vacMD	MDV	vacMD	MDV
9526	-[C]	-	+[D]	++	-	++	[E]											
9527	-	-	+	++	-	++	-	++										
9529	-	-	-	-	+	++	-	++	-	+++								
9543	-	-	-	-	-	-	-	-	-	-								
9545	-	-	-	-	-	-	-	+	-	+++								
9547	-	-	+	++	-	++												
9612	-	-	-	+	-	+++	-	+++										
9613	-	-	+	++	+	++	+	++	-	++								
9618	-	-	+	-	++	-	+	-	-	-								
9619	-	-	-	-	++	-	+	-	+	+								
9625	-	-	-	-	-	++	-	++	-	++								
9629	-	-	-	-	-	++	-	+++										
9633	-	-	-	-	-	++	-	++	-	+++								

Contacts[F]	Day 6		Day 13		Day 20		Day 27		Day 34		Day 43		Day 50		Day 56		Day 68	
Bird #	mRFP	eGFP	mRFP	eGFP	mRFP	eGFP	mRFP	eGFP	mRFP	eGFP	mRFP	eGFP	mRFP	eGFP	mRFP	eGFP	mRFP	eGFP
9539	ND[G]	ND	ND	ND	ND	ND	-	-	-	-	-	-	-	-	-	-	-	-
9542	ND	ND	ND	ND	ND	ND	-	-	-	-	-	-	-	-	-	-	-	-
9548	ND	ND	ND	ND	ND	ND	-	-	-	-	-	-	-	-	-	-	-	-
9603	ND	ND	ND	ND	ND	ND	-	-	-	-	-	-	-	-	-	-	-	-
9621	ND	ND	ND	ND	ND	ND	-	-	-	-	-	-	-	-	-	-	-	-
9622	ND	ND	ND	ND	ND	ND	-	-	-	-	-	-	-	-	-	-	-	-
9627	ND	ND	ND	ND	ND	ND	-	-	-	-	-	-	-	-	-	-	-	-
9628[H]	ND	ND	ND	ND	ND	ND	-	-	-	-	-	-	-	-	-	-	-	++
9647	ND	ND	ND	ND	ND	ND	-	-	-	-	-	-	-	-	-	-	-	-

[A]Birds experimentally infected with green MDV-NT and red vacMD-NT.
[B]Days after experimental infection.
[C]Negative for infection based on fluorescence (see Fig. S1).
[D]Relative level of infection of feathers based on fluorescence (see Fig. S1).
[E]Blacked out boxes indicate birds were euthanized due to clinical MD symptoms and no longer monitored.
[F]Contact chickens housed with experimentally infected birds to monitor natural infection.
[G]ND=not done.
[H]Bird #9628 was positive for eGFP at Day 68 (see Fig. S2).
